# A Systematic Investigation of Differential Effects of Cell Culture Substrates on the Extent of Artifacts in Single-Molecule Tracking

**DOI:** 10.1371/journal.pone.0045655

**Published:** 2012-09-25

**Authors:** Laura C. Zanetti-Domingues, Marisa L. Martin-Fernandez, Sarah R. Needham, Daniel J. Rolfe, David T. Clarke

**Affiliations:** Central Laser Facility, Science and Technology Facilities Council, Research Complex at Harwell, Rutherford Appleton Laboratory, Harwell Oxford, Didcot, United Kingdom; University of South Florida College of Medicine, United States of America

## Abstract

Single-molecule techniques are being increasingly applied to biomedical investigation, notwithstanding the numerous challenges they pose in terms of signal-to-noise ratio issues. Non-specific binding of probes to glass substrates, in particular, can produce experimental artifacts due to spurious molecules on glass, which can be particularly deleterious in live-cell tracking experiments. In order to resolve the issue of non-specific probe binding to substrates, we performed systematic testing of a range of available surface coatings, using three different proteins, and then extended our assessment to the ability of these coatings to foster cell growth and retain non-adhesive properties. Linear PEG, a passivating agent commonly used both in immobilized-molecule single-molecule techniques and in tissue engineering, is able to both successfully repel non-specific adhesion of fluorescent probes and to foster cell growth when functionalized with appropriate adhesive peptides. Linear PEG treatment results in a significant reduction of tracking artifacts in EGFR tracking with Affibody ligands on a cell line expressing EGFR-eGFP. The findings reported herein could be beneficial to a large number of experimental situations where single-molecule or single-particle precision is required.

## Introduction

Since they were first described around 20 years ago, there has been a near-exponential growth in the use of single-molecule techniques for the investigation of biological systems and processes [1]. This is expected to continue as single-molecule methods move out of specialist physics laboratories and find more applications in the biomedical sciences. Single-molecule techniques can essentially be divided into two areas: detection, manipulation, and force measurement using probe microscopy and optical tweezers [2], and techniques that use fluorescence imaging and spectroscopy [3]. Broadly speaking, fluorescence-based methods can be divided into measurements on fluorescent molecules in solution, those on immobilized fluorescent molecules, and measurements on fluorescent molecules in cultured cells. These measurements can be used to investigate stoichiometry, inter- and intramolecular interactions, and molecular conformation. For example, single-molecule fluorescence methods such as nanometer-localized multiple single-molecule fluorescence (NALMS) [4] and fluorescence imaging with one nanometer accuracy (FIONA) [5], are able to provide information on molecular localization and separation, whilst Förster resonance energy transfer (FRET) can be employed to determine the distance between single molecules in the 1–8 nm range [6]. The principle of single-molecule localization also lies behind sub-diffraction limit imaging techniques such as stochastic optical reconstruction microscopy (STORM) [7] and photoactivation localization microscopy (PALM) [8].

The measurement of fluorescence from single molecules is challenging; the signals are weak, and fluorescent molecules suffer from effects such as photobleaching and blinking. In cells, autofluorescence from many molecules contributes background noise. A particular issue that needs to be addressed is that of non-specific binding of fluorescent molecules to the substrate on which the sample is held. Glass substrates are typically used for all types of single-molecule fluorescence measurements, because of their optical transparency, the ability to immobilize molecules on their surface, and their suitability for the growing of cells [1]. Unfortunately, the characteristics which make them suitable for molecular immobilization and cell growth also mean that they readily bind fluorescent molecules such as labelled proteins non-specifically. Non-specific binding contributes spurious fluorescence signals that cannot be readily distinguished from the signals from correctly immobilized molecules, or from fluorescent labels in cells. The presence of large numbers of non-specifically bound fluorescent molecules also makes it more difficult for detection algorithms to successfully locate molecules of interest. In order to address this problem, it is common to passivate glass substrates by coating them with a material that does not interfere with optical transmission or cell growth, but minimizes non-specific binding.

A number of approaches have been taken to passivate the surfaces of glass coverslips for the prevention of non-specific binding. Most simply, proteins or protein-like molecules are added to the surface, so that they might bind non-specifically and block sites that might otherwise be available for non-specific binding of fluorescent protein molecules. Proteins used for this purpose include bovine serum albumin (BSA), fibronectin, laminin, collagen, Poly-L-Lysine, and fetal calf serum (FCS), which contains a mixture of proteins. An alternative approach has been to covalently bind molecules to the glass, creating a monolayer that is resistant to non-specific binding. The most commonly used molecule for this purpose is polyethylene glycol (PEG), in either a linear or branched form [6]. Cells cannot adhere to pure PEG layers, so for single-molecule experiments in cells layers must be supplemented with molecules that allow the cells to bind. RGD peptides, that mimic cell adhesion proteins [9], have been used for this purpose. Finally, a recent publication describes a hybrid approach, using PEG-BSA nanogels [10] for surface passivation. There are a number of studies in the literature that describe the use of individual coatings against non-specific binding of particular molecules [11]–[13] but, surprisingly, a comprehensive comparative study of the effectiveness of commonly used passivation treatments has not been published. Here we describe a comparative study of 10 glass coverslip treatments for single-molecule microscopy. For non-cell experiments we investigated the non-specific binding of three proteins: Human epidermal growth factor (EGF), anti-human epidermal growth factor receptor 2 (HER2) Affibody [14], [15], and hen egg white lysozyme (HEWL), each labelled with three different fluorescent probes. For cell culture experiments, we studied the effect of surface treatments on non-specific binding of anti-human epidermal growth factor receptor (EGFR) Affibody labelled with two different fluorescent probes. We show the effects of non-specific binding on single-molecule tracking experiments, and demonstrate the relative effectiveness of the different surface treatments.

## Results

### Non-specific Binding of Probes to the Glass Depends Predominantly on the Dye

**Figure 1:**
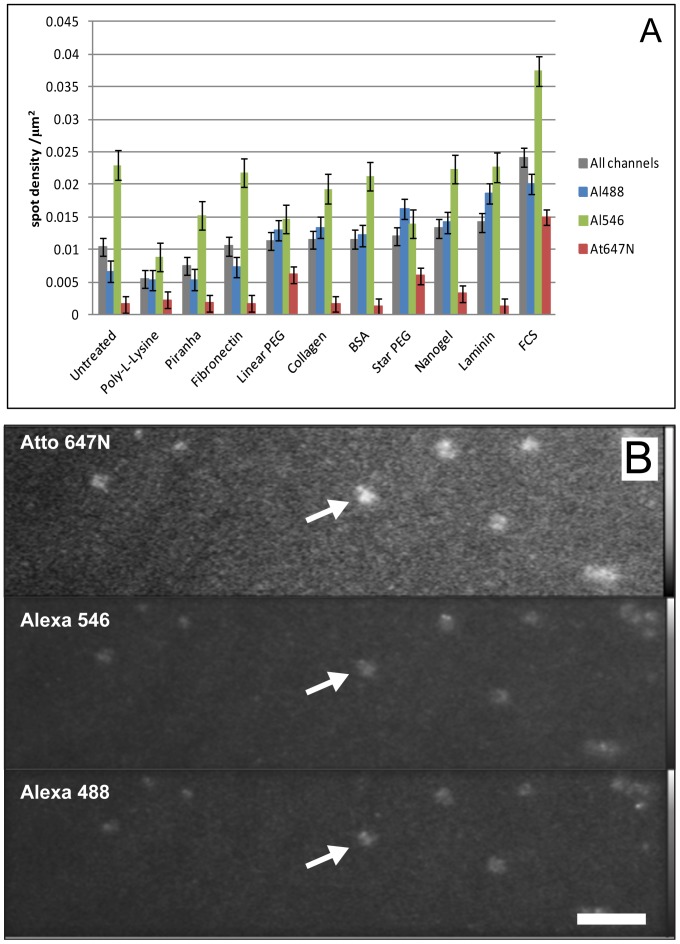
Background fluorescence of treated surfaces. A) Mean spot density/µm^2^ histograms for each surface treatment before exposure to fluorescently-labelled protein (grey columns represent the average calculated on all three channels - each data point corresponds to mean ± SEM from 135 areas, coloured columns represent the averages for each single channel – each data point corresponds to mean ± SEM from 45 areas). B) TIRF fluorescence image of star PEG-treated dish, showing patches of background fluorescence in all detection channels (example arrowed) (bar 8 µm).

We investigated 10 commonly used surface passivation treatments. For single-molecule imaging it is important that surface treatments do not increase the level of background fluorescence from the substrate. Background fluorescence can take two forms; either additional single-molecule spots or diffuse background haze. Fig. 1A compares both individual and the mean spot density for all the channels observed for untreated glass with that of treated glass, before the addition of fluorescent protein. The figure shows comparable mean spot densities for all the treatments, although the density with FCS appears to be marginally higher. This would be expected given that serum contains a cocktail of molecules, some of which are expected to be fluorescent. Also, for all coatings tested, spot density levels appear higher for 488 and 546 channels compared with the far-red channel. Higher levels of background fluorescence haze were seen with both FCS and BSA. Background fluorescence was also observed for star PEG. Five out of the 9 dishes tested showed bright streaks and patches of autofluorescence in all detection channels (example shown in Fig. 1B).

**Figure 2:**
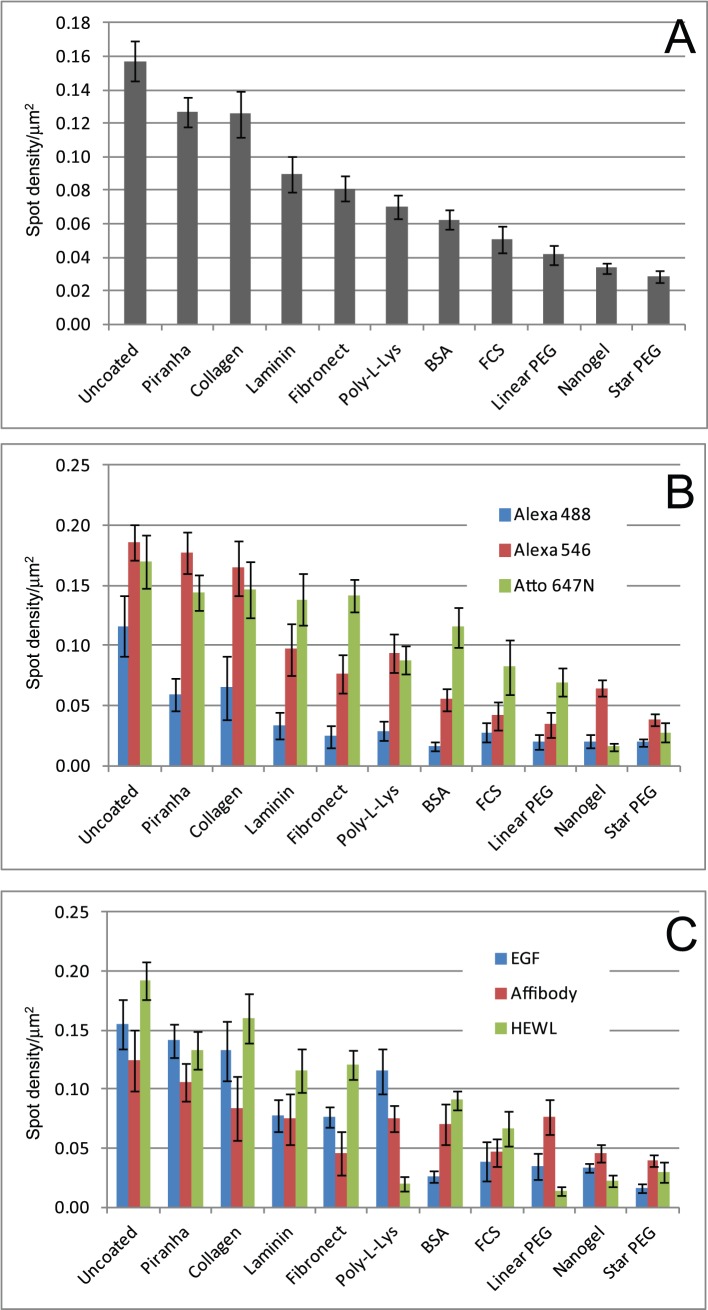
Fluorescent spot densities of treated surfaces after exposure to labelled proteins. **A)** Histogram showing average spot densities/µm^2^ for each surface treatment, after incubation with fluorescently-labelled proteins for 1200 seconds. Each data point corresponds to mean ± SEM from 135 areas, data from all protein-fluorophore combinations being averaged for each treatment. B) Average spot densities/µm^2^ for each fluorophore (each data point corresponds to mean ± SEM from 45 areas, 3 different proteins). c) Average spot densities/µm^2^ for each protein, each data point corresponds to mean ± SEM from 45 areas, 3 different fluorophores).

The relative efficacy of the coatings in preventing non-specific binding of proteins was determined by measuring the density of single-molecule fluorescent spots, detected using Bayesian segmentation [16], after exposure of the treated surfaces to fluorescently labelled proteins. Protein and coating characteristics are detailed in Table 1. Fig. 2 shows the average density of single-molecule fluorescent spots recorded for each treatment. The totals displayed in Fig. 2a show the general trends observed. As expected, untreated glass has the highest level of non-specific binding. Vigorous cleaning with piranha solution only marginally reduces the level of binding. Moderate blocking of non-specific binding is observed for protein-based treatments, collagen being the least effective and FCS and BSA the most effective. Best blocking of non-specific binding is achieved by the PEG-based treatments, with nanogel and star-PEG [17] being slightly more effective than linear PEG.

**Table 1:** Characteristics of ligands, coatings, and fluorophores.

	Mw (Da)	pI	GRAVY^1^	Aliphatic index^2^	Net charge at pH 7
**Ligand**					
EGF	6,045	4.69	−0.506	55.09	−2.2
HEWL	14,313	9.32	−0.472	65.12	7.7
HER2 Affibody	6,736	8.9	−0.625	84.58	2
HER1 Affibody	13,860	4.65	−0.507	79.68	−5
**Coating**					
Poly-L-Lysine	variable (150,000–300,000)	9.6	−3.9	0	poly-cation
Fibronectin	269,110	5.28	−0.513	69.06	−66
Laminin^3^	∼700,000	∼5.3	∼−0.5	∼70	−227
Collagen^4^	∼300,000	∼7.8	−0.261 to 0.919	∼40 to 80	35
BSA	66,433	5.6	−0.475	76.14	−17
FCS	mixture of proteins	variable	variable	variable	variable
Linear PEG	5,000	–	–	–	0
Star PEG	10,000	–	–	–	0
Nanogel	mixture of Star PEG and BSA	–	–	–	–
**Fluorophore**					
Alexa Fluor 488	643	–	–	–	−2
Alexa Fluor 546	1,079	–	–	–	−2
Atto 647N	868	–	–	–	1

1 GRAVY is the grand average of hydropathicity; more negative values indicate greater hydrophilicity [58].

2 Aliphatic index; the relative volume occupied by aliphatic side chains [59].

3 Laminin values taken as average of α1, β1, and γ1 chains.

4 Collagen values representative of the range of collagen types as commercially prepared dishes do not specify type. – indicates not applicable or not known.

The effectiveness of blocking of non-specific binding is also dependent on the protein in use, and on the fluorescent dye with which it is labelled. Fig. 2B and C show the average densities of fluorescent spots, grouped by dye molecule and by protein, respectively. These data show that the effect of the dye is, in most cases, significantly greater than the effect of the protein. Proteins labelled with Alexa 488 show lower levels of non-specific binding than proteins labelled with the other dyes, with most of the surface treatments showing similar levels of effectiveness. Alexa 546 and Atto 647N-labelled proteins show similar degrees of non-specific binding, although there are some differences in the effectiveness of surface treatments for the different dyes. For example, linear PEG works well with Alexa 546 and less well with Atto 647N, while the reverse is true for Nanogel. Differences in non-specific binding between different proteins are less pronounced. The better surface treatments, particularly linear PEG, appear to be marginally less effective against Affibody binding. Some specific effects can also be observed. For example, poly-L-lysine prevents binding of HEWL very effectively, but performs poorly with EGF, but the opposite is observed with BSA treatment. This would be expected given the expected net charge of the proteins and coats.

**Figure 3:**
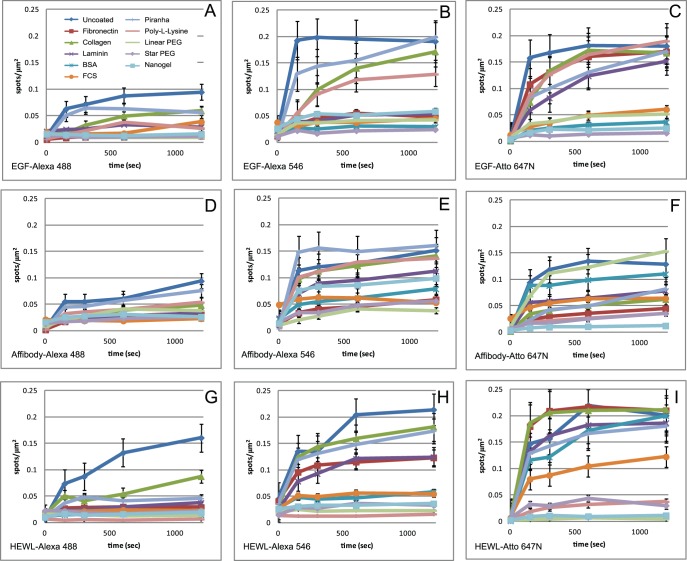
Fluorescent spot density/µm^2^ plots for treated surfaces exposed to the following labelled proteins. A) EGF-Alexa 488, B) EGF-Alexa 546, C) EGF-Atto 647N, D) anti-HER2 Affibody-Alexa 488, E) anti-HER2 Affibody -Alexa 546, F) anti-HER2 Affibody -Atto 647N, G) HEWL C-Alexa 488, H) HEWL C -Alexa 546 and I) HEWL C -Atto 647N, incubated on differently coated glass surfaces for 150, 300, 600 and 1200 seconds at room temperature. Each datapoint corresponds to mean ± SEM of 15 areas acquired from 3 independent samples.

Detailed data on the levels of non-specific binding for all the protein conjugates are shown in Fig. 3. This figure also shows the effect of length of incubation on non-specific binding. As expected, levels of non-specific binding increase with longer incubation times. In general, the individual proteins follow the trends observed in the average data shown in Fig. 2. Some anomalous behaviours are observed, for example Affibody-Atto 647N shows relatively high levels of non-specific binding to linear PEG-treated glass, which is in general one of the more effective treatments. However, it is still reasonably effective with this protein if incubation times are kept short.

### Non-specific Binding of Proteins to Glass can Cause Artefacts when Tracking Molecules on Cell Membranes

**Figure 4:**
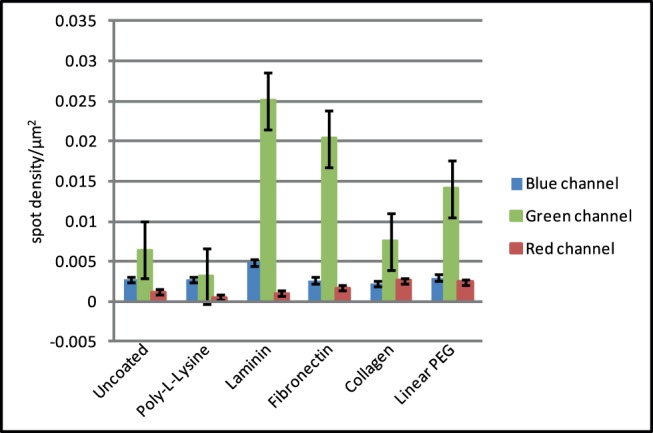
Mean spot density/µm^2^ histograms of background fluorescence of wt Cho cells seeded on different substrates (each data point corresponds to mean ± SEM from at least 15 areas).

Non-specific binding of fluorescent labels to the glass substrate is also problematic for single-molecule measurements on cultured cells. Adherent cells are not completely flat on the substrate and affibodies are able to access a significant area between adherent cells and the glass, particularly when non-confluent cells are used. This permits labelling of receptors in the basal membrane, but also allows non-specific binding to the glass, resulting in fluorescent spots that are undistinguishable from membrane-bound labels.We have investigated the effectiveness of surface treatments against non-specific binding of fluorescent proteins in the presence of cultured cells. These experiments were performed using Chinese hamster ovary (CHO) cells expressing an EGFR-eGFP fusion protein, incubated with anti-EGFR Affibody [18] conjugated with Alexa 546 and Atto 647N. Cells were imaged in a 3 colour TIRF imaging system and single fluorescent molecules were tracked separately in each channel. The eGFP channel acts as a control, as the only fluorescent single molecules detected by the system are those expressed by the cells and localised in the plasma membrane, without the introduction of an additional label. Cell fluorescence contributed by EGFR-eGFP molecules localised in trafficking vesicles or internal membranes is not detected as single-molecule spots, but rather as a diffuse haze. Autofluorescence of the parental cell line was assessed by imaging unlabelled cells, seeded on the same substrates used in the main experiment, using all three lasers. Fig. 4 shows that the background signal of the cells is in the same range as that of the unlabelled dishes (Fig. 1A), showing that the main contribution stems from impurities of the substrate rather than from cell autofluorescence.

**Figure 5:**
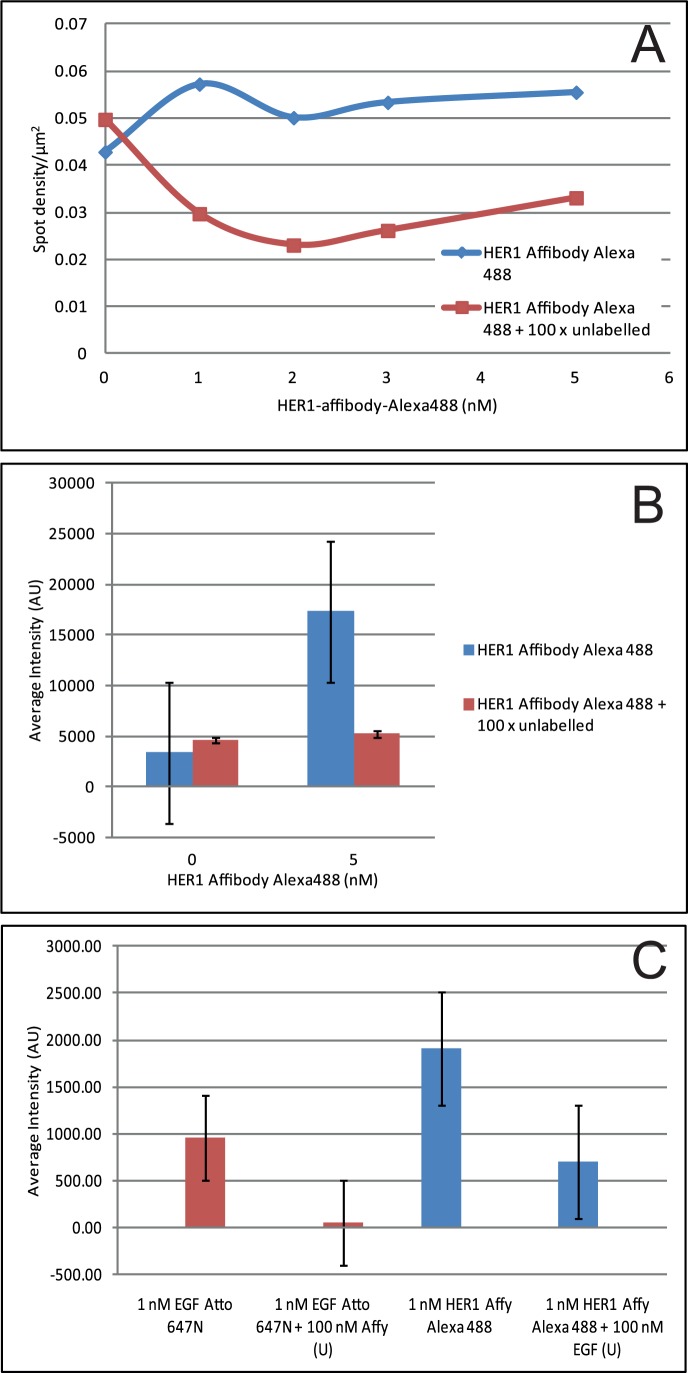
Determination of level of non-specific binding of labelled proteins to cells. A) Spot density/µm^2^ plot for HER1 Affibody Alexa488 on T47D cells in presence (red) or absence (blue) of 100x excess unlabelled HER1 Affibody as determined by fluorescent single-molecule detection. B) Average intensity histogram of HER1 Affibody Alexa488 on A431 cells in presence (red) or absence (blue) of 100x excess unlabelled HER1 Affibody as determined by confocal imaging. C) Average intensity histogram of HER1 Affibody Alexa488 (blue) and EGF Atto 647N (red) on A431 cells in presence or absence of 100x excess unlabelled competitor.

The specificity of the probe and therefore the extent of non-specific binding to the cells was assessed both by confocal and single-molecule microscopy. A431 cells, which overexpress EGFR were used for confocal experiments for higher intensity and T47D cells, which express low levels of EGFR, were used in single-molecule experiments to enable detection of individual spots. As shown in Fig. 5A and 5B, competing the labelled species with 100x unlabelled Affibody reduced the labelling to background levels, which should rule out non-specific interactions with the membrane. The specificity of HER1 Affibody for the EGFR receptor was assessed by competing a labelled species (EGF or Affibody) with 100x concentration of the other species. Cross-competition, already demonstrated for Z:EGFR955 [19], a close sequence relative of the commercial Affibody used in our experiments, reduced the signal to background levels for both species (Fig. 5C).

**Figure 6:**
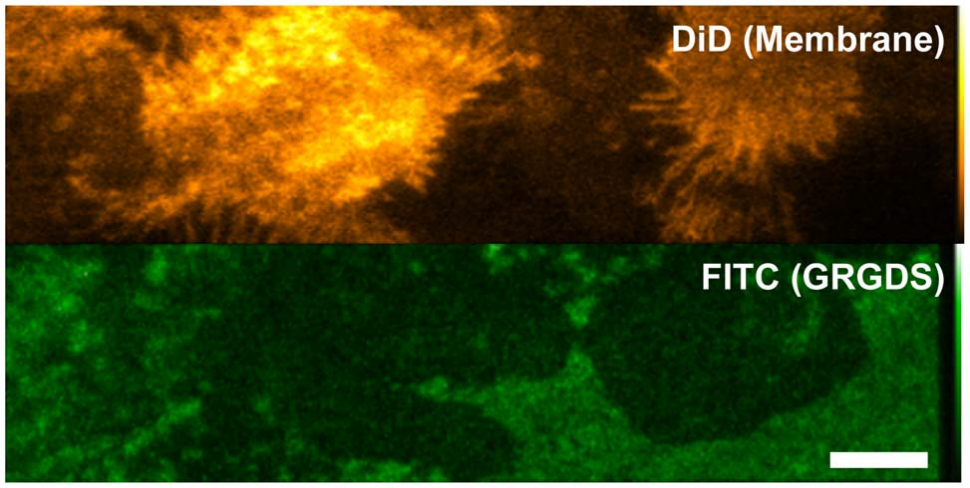
TIRF image of PEG-treated glass doped with fluorescent GRGDS peptide and T47D cells treated with DiD membrane probe to highlight membrane protrusions and membrane-glass contact areas (bar 8 µm).

Assessment of non-specific binding to the substrate vs specific binding of the Affibodies to EGFR was made by monitoring the diffusion coefficients of the fluorescent spots observed in the eGFP, Alexa 546, and Atto 647N channels. In the absence of non-specific binding, all channels would be expected to show similar rates of diffusion, with similar numbers of non-mobile molecules. As non-specifically bound molecules should be immobile, higher levels of non-mobile molecules in the Alexa 546 and Atto 647N channels with respect to the eGFP channel would indicate the presence of non-specific binding. We investigated a number of surface treatments typically used in single-molecule cell experiments: 0.01% poly-L-lysine, 25 µg/ml fibronectin, 25 µg/ml laminin, collagen, 1% BSA, and linear PEG. As cells do not adhere to linear PEG, the coating was doped with GRGDS peptide [9]. In order to confirm that GRGDS peptide was incorporated into the PEG layer, we also prepared and imaged layers containing FITC-labelled GRGDS (Fig. 6, see Materials and Methods for details). We did not use star PEG or nanogel for cell experiments because of higher levels of background autofluorescence.

**Figure 7:**
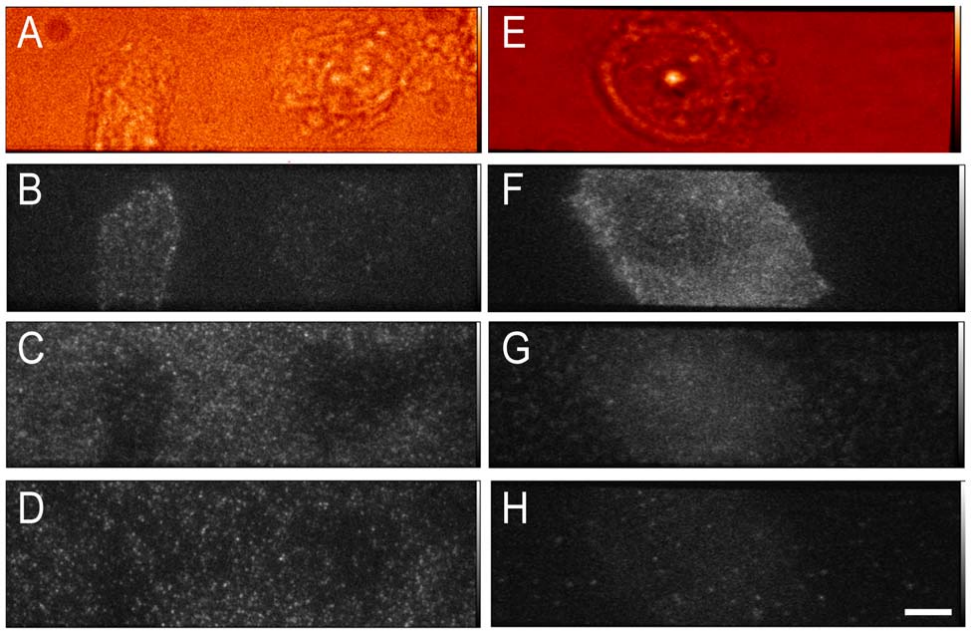
Representative images of cells exposed to labelled proteins. Panel A–D: representative images of CHO-EGFR-eGFP cells grown on uncoated glass. Whitelight (A), anti-EGFR Affibody Atto 647N (B), anti-EGFR Affibody Alexa 546 (C), and EGFR-eGFP (D). Panels E–H: representative images of CHO-EGFR-eGFP cells grown on linear-PEG +0.4 mM GRGDS peptide-coated glass. Whitelight (E), Anti-EGFR Affibody Atto 647N (F), anti-EGFR Affibody Alexa 546 (G), and EGFR-eGFP (H) (bar 8 µm).

**Figure 8:**
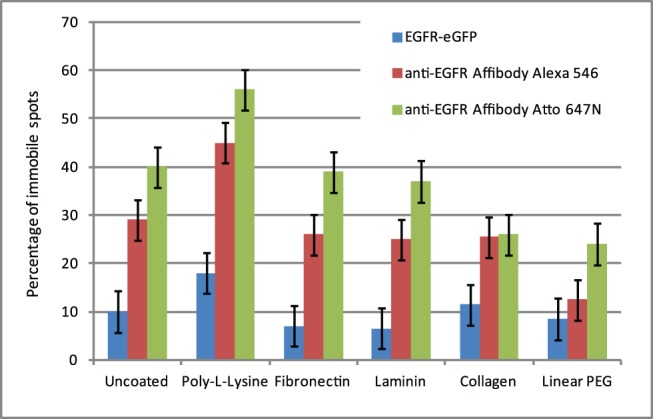
Histogram showing percentage of tracks with diffusion coefficient falling in the D = 0 bin of the D distribution histogram in the three acquisition channels on CHO-EGFR-eGFP cells grown on differently coated glass surfaces and labelled with anti-EGFR Affibody Alexa 546 and Atto 647N for 15 minutes at 37°C. Each datapoint corresponds to mean ± SEM of 15 areas acquired from 3 independent samples.

**Figure 9:**
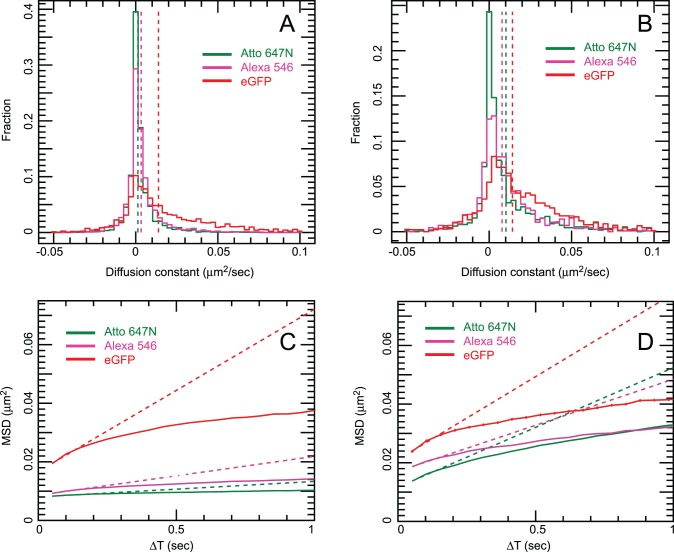
Side-by-side comparison of mean squared displacement (MSD) curves and diffusion coefficient (D) histograms from CHO-EGFR-eGFP cells grown on uncoated glass vs. linear-PEG+0.4 mM GRGDS-coated glass. Data were plotted from at least 15 areas acquired from 3 independent samples. Each MSD value comes from at least 6500 (ranging up to 300,000) individual separations, resulting in very small standard error in the MSD. Error bars are plotted but too small to be visible. Panels A (uncoated) and B (linear PEG + GRGDS): diffusion coefficient histogram of tracked spots. EGFR-eGFP (red), anti-EGFR Affibody Alexa 546 (magenta), anti-EGFR Affibody Atto 647N (green). Dotted lines show the mean D coefficient extrapolation. Panels C (uncoated) and D (linear PEG + GRGDS): Mean Square displacement plot. EGFR-eGFP (red), anti-EGFR Affibody Alexa 546 (magenta), anti-EGFR Affibody Atto 647N (green). Dotted lines show the mean D coefficient extrapolation.

Cells grew well on both uncoated and linear PEG/GRGDS substrates, and typical images are shown in Fig. 7. The motion of fluorescent spots was tracked and percentages of immobile fluorescent spots are plotted in Fig. 8. In general, there was a higher level of non-specific binding for Affibody labelled with Atto 647N than with Alexa 546. Both collagen and linear PEG treatment significantly reduced binding for Atto 647N Affibody, only linear PEG treatment resulted in significantly lowered binding for Alexa 546 Affibody. None of the other treatments significantly reduced non-specific binding, and poly-L-lysine treatment increased binding for Affibodies labelled with either dye. The effect of non-specific binding on tracking experiments can be seen in Fig. 9, which compares the motion of anti-EGFR Affibody labelled with Alexa 546 and Atto 647N on CHO cells cultured on untreated glass, and glass treated with linear PEG doped with GRGDS peptide. Comparison of the diffusion coefficient distribution histograms in Fig. 9 A (untreated) and B (linear PEG) reveals that the motion of Affibody in the latter experiment is closer to the motion of the reference eGFP; for uncoated glass, the distribution of Affibody mobility is skewed towards slower moving and stationary molecules. Similarly, in the mean squared displacement plots shown in Fig. 9 C (untreated) and D (linear PEG), displacements in the linear PEG experiment are much closer to the displacements in the reference channel than for untreated glass. Varying offsets at t = 0 are observed in the MSD plots. These are likely to be the result of different levels of spot localization errors, a function of the signal-to-noise of individual data sets. The MSD plots for EGFR-eGFP and for the two affibody probes in fig. 9D display a profile compatible with anomalous subdiffusion, which is expected and can be explained in the framework of the membrane-skeleton picket-fence model [20] by temporary corralling of the receptor by the membrane cytoskeleton or by other transmembrane proteins bound to the cytoskeleton. Additional compartmentalisation can be provided by EGFR association with lipid rafts [21], which have been shown to act as transient confinement zones [22].

## Discussion

The data described above confirm that non-specific binding is an important issue for single-molecule experiments. We observe significant levels of non-specific binding both in the presence and absence of cells. For non-cell experiments, it is important that only data from specifically immobilized molecules are observed, as uncontrolled binding to the substrate through non-specific interaction can result in denaturation of the molecules [23], and therefore non-representative results. For cell experiments, we have demonstrated that high levels of non-specific binding can significantly affect measured diffusion coefficients and mean squared displacements, important parameters for tracking experiments. This problem cannot be eliminated by discounting data from immobile molecules, as it has been reported that transmembrane receptors, and in particular EGFR, can be immobilized or slowed down for variable periods of time when they are actively engaged in signalling [24]–[27], or even in the resting state [25], [28]. Immobilisation of EGFR molecules has been observed in different cell lines and with different techniques such as FRAP [28], single-fluorescent-molecule tracking [24], [25], [27] and single-particle video-rate tracking [26]. Possible mechanisms underlying the immobilisation of EGFR molecules are likely to be manifold. EGFR is known to bind to actin filaments [29] and indeed the depolymerisation of actin can alter the diffusional behaviour of the receptor [24], [28], [30]. EGFR is also associated with lipid rafts (reviewed in [31] and [32]), which regulate its activation and diffusion. Disruption of caveolae [25] and cholesterol depletion [24], [30] are also able to alter the immobile fraction of EGFR in the membrane, at least in some cell systems. Another candidate for receptor immobilisation is the galectin lattice that cross-links glycoproteins on the extracellular side of the membrane. The disruption of this lattice leads to inceased mobility of EGFR [28]. Finally,the immobilisation of active receptors is linked to the activity of the tyrosine kinase domain [27] The presence of an immobile fraction of EGFR is confirmed in our experiments, in which we observe a percentage (5–15%, variable but still well within observations in the literature [25], [26], [28]) of immobile molecules in the eGFP reference channel. This variability can be due to differential activation of adhesion receptors by the substrates: fibronectin, laminin, collagen and GRGDS peptide activate different complements of integrin subunits (reviewed in [33], [34]), while Poly-L-Lysine is supposed to act by favouring electrostatic interactions with the cell membrane. The differential effect of different substrates on cell behaviour has been reported in the biomaterials field [35]–[40] and in this case the effect can be compounded by the fact that integrin signalling is known to interweave with EGFR signalling (reviewed in [41]). Since receptor immobilization might be a feature of signalling, it is essential to keep the levels of non-specific binding to a minimum for all single-molecule experiments.

Our systematic study of the effects of various surface treatments shows that the levels of non-specific binding are the result of the interaction of three factors; the treatment used, the protein, and the fluorescent dye with which the protein is labelled. We consistently observed lower levels of non-specific binding for protein labelled with Alexa 488, while Atto 647N-labelled proteins showed the highest levels of binding. This is probably caused by the different charge and hydrophobicity characteristics of the dyes; Alexa 488 is negatively charged and hydrophilic, whereas Atto 647N has a positive charge and is hydrophobic [42]–[44]. High levels of non-specific binding of Atto 647N conjugates have been reported previously [42]. Careful selection of dye is therefore an important starting point when planning single-molecule experiments. If a choice of dyes is available, it would be advisable to perform test experiments and select the dye that results in the lowest levels of non-specific binding. However, other considerations such as appropriate spectral characteristics and photostability will affect dye choice. For example, although we observe higher levels of non-specific binding with Atto 647N, this dye is particularly stable and may still be the best choice for long-term tracking of single molecules [45].

Choice of protein is usually much more restricted than choice of fluorescent label. Obviously the protein of interest is the protein that must be used, whatever its binding characteristics, although there may sometimes be a choice available amongst, for example, different antibodies against the target protein. However, this is the exception so in most cases the experimenter must select an appropriate surface treatment for the protein in use. We selected three proteins with varying characteristics (EGF: pI 4.5, negatively charged at pH 7.4, mix of hydrophobic and hydrophilic residues; Affibody: pI 9.0, slightly positively charged at pH 7.4, hydrophobic and hydrophilic regions; and HEWL pI 11.3, strongly positively charged at pH 7.4, mainly hydrophilic) and tested them against a range of substrates with different surface change and hydropathy profiles: For a summary of protein and coating characteristics, see Table 1. In general, the relative efficacy of the surface treatments was similar for all proteins, with the PEG-based treatments (linear PEG, star PEG, and nanogel) being the most effective in preventing non-specific binding. However, in some cases the combination of a different coating with a specific protein was particularly effective. For example, poly-L-lysine treatment resulted in very low levels of non-specific binding of HEWL. This is not unexpected, given that both coating and protein are strongly positively charged at the pH used in the experiments, so electrostatic repulsion would be expected to inhibit binding. Conversely, the effectiveness of BSA was limited to EGF. These data show that in some cases careful choice of surface treatment to match the protein in use can be an effective approach.

When the experiment involves measurements in cells, the choice of surface treatment is more complex. The major problem is that coatings which prevent non-specific binding of proteins tend to hinder the interactions that allow cells to adhere to surfaces. Also, adsorption of proteins from the cell culture medium, extracellular proteases and extracellular matrix (ECM) deposition by cells can modify the culture substrate [34]. Modification of substrates by cells, such as matrix degradation by extracellular matrix metalloproteases, may make the subtrates more susceptible to adsorption of spurious proteins, which might explain why coatings such as poly-L-lysine, fibronectin, collagen and laminin, which were moderately effective in preventing non-specific binding in the absence of cells, were ineffective when cells were present. The most effective treatments for non-cell measurements were those based on PEG, and our results with linear PEG confirm this to be the case for cell experiments as well. Growing cells on synthetic biomaterial substrates, such as PEG, is a staple technique of tissue engineering and regenerative medicine. PEG is employed due to its ability to repel non-specific protein adhesion ([46] and reviewed in [47]–[49]). PEG is an uncharged, hydrophilic polymer which displays low toxicity to cells. It is able to undergo extensive hydration in aqueous mediums, by virtue of displaying two hydrogen-bond acceptor groups. This, along with its conformational flexibility, causes a volume restriction effect that hinders protein deposition on PEG layers [50]. The compression of the polymer layer by incoming proteins is unfavorable from a thermodynamic standpoint [51].While these properties and the fact that the polymer is not biodegradable [48], make PEG an ideal and stable reagent for passivation, they also make cell adhesion problematic and anchorage-dependent cell viability low, therefore PEG surfaces and other biomaterials are routinely doped with adhesion peptides and biomolecules derived from ECM proteins and proteoglycans to encourage cells to grow, divide and differentiate [33]. Various adhesion peptides are available, GRGDS being one of the most widely used [34]. In our hands, CHO-EGFR-eGFP were able to grow on Linear PEG functionalized with as little as 0.2 µM GRGDS peptide (data not shown), albeit at the expense of faster apoptosis under serum deprivation conditions during experimental time. Using GRGDS peptide at a concentration of 0.4 mM resulted in a surface that was able to sustain the growth of CHO-EGFR-eGFP, yielding confluent monolayers of cells with a normal morphology (flat, polygonal, and elongated, compared with a rounded appearance and ruffled membrane for unhealthy cells) and EGFR-eGFP expression level. GRGDS peptide is known to bind to αvβ3, α5β1 and αvβ5 integrins [9], [34]. Different peptides, such as laminin-derived YIGRS and IKVAV [34], [52], [53] could be better suited to cell lines expressing a different complement of adhesion receptors.

### Conclusions

Single-molecule techniques are increasingly popular in the biomedical field and are being used to investigate a wealth of biological questions. Non-specific binding of fluorescent single molecules is a major problem for single-molecule experiments. Our systematic investigation of surface passivation treatments demonstrates that non-specific binding can be minimized by careful selection of fluorescent labels, and by tailoring the surface treatment to suit the type of molecule being investigated. Treatments based upon PEG appear to give the best results overall, although care must be taken to use high purity materials to minimize background fluorescence.

Linear PEG seems a good option for functionalizing glass surfaces for single-molecule experiments in cells, as it can repel different proteins characterized by different MW and pI and retains its properties even after cells are grown on it and despite their modifying effect, whereas other biological substrates of comparable efficacy in cell-free settings might be affected by ECM degradation/deposition processes, as evidenced by our live-cell tracking experiments. A wealth of adhesive peptides can be combined with linear PEG in order to facilitate adhesion of different cell strains and, while 0.4 mM GRGDS was a good solution for our cell model, it is probable that case-by-case optimization of adhesive cocktails will be needed.

## Materials and Methods

### Surface Passivation

Glass-bottom cell culture dishes (MatTek Corporation) were used for all surface treatments. Details of surface passivation treatments were as follows:

#### “Piranha” cleaned dishes

150 µl of concentrated sulphuric acid were mixed with 50 µl of 30% w/v hydrogen peroxide *in situ* on the cover slip. The solution was left for 15 minutes at room temperature and the dishes then rinsed with copious amounts of deioinsed water. Dishes were allowed to dry and stored at room temperature in sealed plastic sleeves.

#### Polyethylene glycol

Dishes were first cleaned with piranha solution as described above. Dishes were then treated with 4-aminopropyl triethoxysilane (APTES; Sigma Aldrich), as follows. A 2% v/v solution of APTES in water was made up immediately prior to treatment. The APTES solution was added to the dishes, completely covering the glass, and dishes were incubated for 15 minutes at room temperature. Dishes were then rinsed extensively with deionized water and allowed to dry. Dishes were left overnight at room temperature before the next stage. PEG solutions were prepared immediately before use. Either 8-arm PEG-vinyl sulfone, MW 10K “star-PEG” (Creative PEGWorks), or PEG-succinimidyl valerate, MW 5K “linear PEG” (Laysan Bio), were dissolved at a concentration of 200 mg/ml in filtered sodium bicarbonate buffer, pH 8.5. The solution was added to the dishes, and incubated for 3 hours at room temperature. Dishes were then rinsed thoroughly with deionized water, allowed to dry, and stored at 2–8°C for use within 2–4 weeks. In dishes to be used for cell culture, GRGDS peptide (Anaspec) was added to the PEG solution at concentrations of 0.2 µM or 0.4 mM. FITC-labelled GRGDSP peptide (Anaspec) was used at concentrations of 0.2–200 µM. Dishes were stored in sealed plastic sleeves at 2–8°C and used within six weeks of coating.

#### PEG-BSA nanogels

PEG-BSA nanogels were prepared using 8-arm PEG-vinyl sulfone and BSA, as described by Tessler *et al.* [10]. Dishes were first cleaned with piranha solution and treated with APTES as described above. 10% w/v nanogel in PBS was added to the dishes, and they were incubated for 1 hour at 37°C. The dishes were then washed in PBS and incubated for 1 hour at 37°C with 50 mg/ml BSA in PBS. The dishes were exposed to 1 M Tris, pH 8.0 for 15 minutes at room temperature to quench unreacted vinyl sulfone groups. Finally, dishes were washed with PBS. Dishes were filled with PBS to prevent layer desiccation and stored at 2–8°C for use within 2–4 weeks.

#### Poly-L-lysine

Dishes were first cleaned with piranha solution, as described above. 0.01% w/v poly-L-lysine in deionized water was added to dishes, covering the surface of the glass. Dishes were incubated for 3 hours at room temperature, and the poly-L-lysine solution was then aspirated off, and the dishes stored at room temperature for use within 2–4 weeks.

#### Bovine serum albumin

A solution of 1% w/v of Bovine Serum Albumin (Sigma) in PGBS was prepared and sterile filtered. Enough solution to cover the glass was added to the dishes and incubated at room temperature for 1 hour. BSA solution was then aspirated off and dishes were stored at 4°C for use within 2–4 weeks.

#### Fetal calf serum

Pure Fetal Calf Serum (Gibco) was added to the glass coverslips and incubated at room temperature for 1 hour. FCS was then aspirated off and dishes were stored at 4°C for use within 2–4 weeks.

#### Laminin

25 µg/ml laminin (Sigma) in PBS was added to dishes, covering the surface of the glass. Dishes were incubated for 2 hours at room temperature, and the laminin solution was then aspirated off, and the dishes stored at 2–8°C for use within 2–4 weeks. For cell culture experiments, dishes were rinsed with culture medium before plating cells.

#### Fibronectin

25 µg/ml fibronectin (Sigma) in PBS was added to dishes, covering the surface of the glass. Dishes were incubated for 45 minutes at room temperature, and the fibronectin solution was then aspirated off, and the dishes stored at 2–8°C for use within 2–4 weeks. For cell culture experiments, dishes were rinsed with culture medium before plating cells.

#### Collagen

Commercially available collagen-coated glass-bottomed dishes were used (MatTek Corporation).

### Assessment of GRGDS Incorporation into PEG Layers

Incorporation of GRGDS peptide into the PEG coating was measured by imaging coatings doped with different concentrations of FITC-labelled GRGDSP peptide. T47D cells (ECACC) were cultured in RPMI 1640 medium and supplemented with 10% FCS, 2 mM L-Glutamine and 1% penicillin/streptomycin (all Invitrogen). Cells were seeded on PEG-coated dishes doped with FITC-RGD, at a density of 3×10^5^ cells/dish, cultured until 70% confluence was reached, then deprived of serum for 2 h and labelled with 5 µM DiD membrane probe (Invitrogen) to assess cell spreading and surface contact. Cells were imaged live on a TIRF microscope. The image in Fig. 6 shows significant levels of FITC fluorescence, demonstrating the presence of the fluorescent peptide in the PEG layer and acceptable cell morphology, with presence of filopodia, protrusions and focal adhesions, which indicate that the cells are able to bind to the peptide embedded in the PEG layer. Reduction of fluorescence in the FITC channel in correspondence with adhesion points might be due to energy transfer between FITC and DiD or to peptide uptake by cells.

### Cell culture

CHO-EGFR-eGFP cells (kind gift of Dr. Donna Arndt-Jovin [54]) were cultured in DMEM without phenol red and supplemented with 10% FCS, 2 mM L-Glutamine, 1% penicillin/streptomycin and 2 ml/l Geneticin (all Invitrogen). Wt Cho and A431 cells were cultured in DMEM without phenol red and supplemented with 10% FCS, 2 mM L-Glutamine and 1% penicillin/streptomycin (all Invitrogen). Cells were plated on uncoated, poly-L-lysine-coated, collagen-coated, fibronectin-coated, laminin-coated or linear PEG/GRGDS-coated glass-bottomed dishes at a density of 10^5^ cells/dish.Cells were rinsed twice with serum-free medium and starved for 2 hours upon reaching 80% confluence to remove serum-derived growth factors which can interfere with probe binding.

### Fluorescent Labelling of Proteins

Alexa 488, Alexa 546 (both Sigma) and Atto 647N (Atto Tec) NHS-ester were conjugated to the N-terminus of murine epidermal growth factor (EGF) in a 1∶1 stoichiometry by Cambridge Research Biochemicals. Anti-EGFR Affibody and anti-HER2 Affibody (both Abcam) were labelled at single cysteine residues in a 1∶1 stoichiometry with Alexa 488, Alexa 546 and Atto 647N maleimide, following the manufacturer’s instructions. Alexa 488, Alexa 546 and Atto 647N NHS-ester were conjugated to the N-terminus of Hen Egg White Lysozyme (Sigma) in a ∼1∶1 stoichiometry as per dye manufacturer’s instructions.

### Cell-free Assessment of Non-specific Protein Binding

Glass-bottomed dishes were imaged (see below) firstly upon addition of PBS solution, in order to ascertain the presence and amount of impurities, taking single-frame images of 5 non-overlapping areas, then PBS was aspirated off and glass dishes were treated with a solution of triply labelled protein (10 nM of each labelled species for EGF and HEWL, 11 nM of each labelled species for anti-HER2 Affibody). Single-frame images of 5 non-overlapping areas were taken at 150 s, 300 s, 600 s, and 1200 s. Experiments were performed in triplicate and spot density/µm^2^ was calculated for each imaged area and logged in GraphPad Prism 5 (GraphPad Software, Inc.). Mean ± SEM of 15 areas was plotted for each data point.

### Cell Labelling

Starved cells were rinsed twice with PBS pH 7.4 pre-heated at 37°C and labelled with 2 nM each anti-EGF Affibody Alexa 546 and Atto 647N for 15 minutes at 37°C. Cells were rinsed twice with PBS pH 7.4 pre-heated at 37°C and promptly imaged as described below. For probe specificity assessment, starved cells were rinsed twice with chilled PBS pH 7.4 and labeled with the appropriate amount of HER1 Affibody or EGF with or without 100x excess unlabelled HER1 affibody or EGF. Cells were rinsed twice with chilled PBS pH 7.4 and fixed in 3% paraformaldehyde (Electron Microscopy Sciences), 0.5% glutaraldehyde (Sigma) for 30 minutes prior to imaging.

### Confocal Data Acquisition and Analysis

Confocal images used to assess HER1 Affibody specificity were acquired using a Nikon, Eclipse Ti microscope equipped with a, Nikon, D-Eclipse C1 scanning unit and a PMC100-1 photomultiplier (Becker & Hickl GmbH). The output from the AOTF of a supercontinuum laser source (Fianium, SC450-4, 40 MHz repetition rate) at wavelength bands 491 nm and 635 nm was used as an illumination source. Average intensity from confocal images was calculated with ImageJ software (NIH) [55] by thresholding the image and converting it into a binary mask, then substracting it from the original image and obtaining the list of pixel intensity frequencies through the Pixelhoover v1 plugin (for 16-bit images) or through the Analyze>Histogram command. Pixel intensity frequencies for different areas of the same sample were imported in Excel spreadsheets, added up and multiplied by their intensity values (1–255), then averaged. Intensity data from at least 30 cells were used for each treatment.

### Single-molecule Data Acquisition

Single- molecule images were acquired using a Zeiss Axiovert TIRF-setup with excitation wavelengths λ = 491 nm (100 mW, Cobolt Calypso), 561 nm (100 mW, Oxxius SLIM), 639 nm (30 mW, PTI IQIC30), as described previously [56]. Field of view of each channel for single-molecule imaging was 80×30 µm. Tracking data of triply labelled cells was acquired at 20 Hz for 30 seconds. At least 15 areas were acquired over three independent replicates for each experimental condition. For probe specificity assessment, data were acquired at 10 Hz for 1 minute. At least 10 areas were acquired for each experimental condition. Images were saved in HDF5 format for subsequent processing using custom-designed software [16].

### Analysis of Tracking Data

All single-molecule time series data were analysed using the multidimensional analysis software described in [16]. Registration transformations were determined but feature detection and tracking was performed independently in each channel. For each time series, cell areas were outlined manually using new functionality in the software in [16]. Single-molecule tracks whose mean positions were in the outlined cell areas were pooled together for cells imaged under the same conditions and their Mean Square Displacement (MSD) curve calculated. MSD was calculated as MSD(Δ*T*) = <|*r_i_*(*T+*Δ*T*)−*r_i_*(*T*)|^2^> where |*r_i_*(*T+*Δ*T*)−*r_i_*(*T*)| is the displacement between position of track *i* at time *T* and time *T*+Δ*T* and the average value is over all pairs of points separated by Δ*T* in each track. Histograms of instantaneous diffusion coefficients (*D*) were calculated by calculating an MSD curve separately for each track, fitting a straight line to the first 3 points of that MSD curve then calculating *D* directly from the gradient *m* of the fit, *D* = *m*/4 (see e.g. [57]). Histograms were then produced using the *D* values for the pooled tracks from the selected cell areas.
